# Parliament and the Profession

**Published:** 1916-05-06

**Authors:** 


### PARLIAMENT AND THE PROFESSION.

### Supply of Poisonous Drugs to Transports.

The President of the Board of Trade has promised to make inquiries into the question of the supply of poisonous drugs to transports. The question was raised by Mr. L. Haslam, who asked whether such drugs were supplied to transports by unqualified chemists; and, if so, whether, in view of the dangers involved owing to this practice, he would cause its discontinuance.

### Travelling Medical Boards.

To a question with regard to the constitution and duties of travelling medical boards, asked by Mr. Prothero, Mr. Tennant replied that the functions of a travelling medical board were quite different from those of a standing medical board; the former placed the men in the prescribed categories, and the latter determined questions of invaliding. He added that no case had been brought to notice in which the recommendations of a travelling medical board had been cancelled by a standing medical board.

### Fees for Medical Examination of Recruits.

The question of the amount of the fee paid to medical men for the examination of recruits was raised by Mr. Joynson-Hicks. He pointed out that in the early period of the war a number of doctors were appointed by recruiting officers to examine recruits at a fee of 2s. 6d. per head, and that during the rush in December, 1915, many doctors gave up their private practice, and, with the help of assistants, working twelve to fifteen hours a day, examined and passed a number of recruits, but had been refused payment of anything more than £2 a day. Mr. Forster admitted that at the beginning of the war the regulation fee was 2s. 6d. per recruit examined, but stated that in February, 1915, the regulation was modified to the effect that where considerable numbers were examined £2 would be the maximum payment for a full day's work.

### Other Matters of Interest.

Small-pox at Salford.—Replying to Mr. Clynes, Mr. Hayes Fisher stated that there had been eight cases of small-pox in Salford during the present year—all in persons over eighteen. Six of them had been vaccinated in infancy but not since, one was unvaccinated, and one case—which was the only fatal one—was doubtful. Of about 40,000 children born in Salford in the six years 1909-1914 about 4,800 had not been vaccinated at the date of the last vaccination officer's returns.

Military Tribunals and Unregistered Dentists.—The attention of the President of the Local Government Board was called by Mr. Raffan to the fact that in several cases members of local tribunals have made remarks of an offensive nature to applicants who pleaded that they were carrying on work as unregistered practitioners of dentistry, and that such remarks injured the applicants in the pursuit of their calling. Mr. Long, in his reply, said he would deprecate the use of offensive remarks towards any applicant, and he was sure his feeling would be shared generally by members of the tribunals.

